# Effects of dietary sulfur amino acid levels on growth performance and intestinal immunity in broilers vaccinated and subsequently infected with coccidiosis

**DOI:** 10.1016/j.psj.2023.102557

**Published:** 2023-02-03

**Authors:** Changqing Li, Jie Chen, Jiajie Wang, Rose Whelan, Daniel E. Bütz, Mitchell D. Ramuta, Wentao Wang, Jiachen Li, Xin Yang, Yanli Liu, Xiaojun Yang, Mark E. Cook, Thomas D. Crenshaw, Zhouzheng Ren

**Affiliations:** ⁎ College of Animal Science and Technology, Northwest A&F University, Yangling, Shaanxi 712100, China; † Evonik Operations GmbH, Hanau-Wolfgang 63457, Germany; ‡ Department of Animal and Dairy Sciences, University of Wisconsin-Madison, Madison, WI 53706, USA

**Keywords:** broiler, *Eimeria*, intestinal immunity, sulfur amino acid, vaccination

## Abstract

Coccidia vaccination is a common practice in the poultry industry. However, research is lacking regarding the optimal nutritional support for coccidia vaccinated broilers. In this study, broilers were vaccinated with coccidia oocyst at hatch and were fed with a common starter diet from 1 to 10 d. On d 11, the broilers were randomly assigned to groups in a 4 × 2 factorial arrangement. Briefly, the broilers were fed one of four diets containing 0.6, 0.8, 0.9, and 1.0% of standardized ileal digestible methionine plus cysteine (**SID M+C**), respectively, from 11 to 21 d. On d 14, the broilers from each diet group were orally gavaged with either PBS (Mock challenge) or *Eimeria* oocysts. Compared to PBS-gavaged broilers and regardless of dietary SID M+C levels, the *Eimeria*-gavaged broilers had 1) decreased gain-to-feed ratio (15–21 d, *P* = 0.002; 11–21 d, *P* = 0.011); 2) increased fecal oocysts (*P* < 0.001); 3) increased plasma anti-*Eimeria* IgY (*P* = 0.033); and 4) increased intestinal luminal interleukin-10 (**IL-10**; duodenum, *P* = 0.039; jejunum, *P* = 0.018) and gamma interferon (**IFN-γ**; duodenum, *P* < 0.001; jejunum, *P* = 0.017). Regardless of *Eimeria* gavage, broilers fed 0.6% SID M+C had decreased (*P*<0.001) body weight gain (15–21 and 11–21 d) and gain-to-feed ratio (11-14, 15-21, and 11-21 d) when compared to those fed ≥ 0.8% SID M+C. *Eimeria* challenge increased (*P* < 0.001) duodenum lesions when the broilers were fed with 0.6, 0.8, and 1.0% SID M+C, and increased (*P* = 0.014) mid-intestine lesions when the broilers were fed with 0.6 and 1.0% SID M+C. An interaction between the two experimental factors was detected on plasma anti-*Eimeria* IgY titers (*P* = 0.022), as coccidiosis challenge increased plasma anti-*Eimeria* IgY titers only when the broilers were fed with 0.9% SID M+C. In summary, the dietary SID M+C requirement for grower (11–21 d) broilers vaccinated with coccidiosis was ranged from 0.8 to 1.0% for optimal growth performance and intestinal immunity, regardless of coccidiosis challenge.

## INTRODUCTION

*Eimeria*-induced coccidiosis is a major concern for the antibiotic-free poultry industry (Kadykalo et al., 2018). An interaction between coccidiosis infection and dietary sulfur amino acid requirements infers that sulfur amino acid requirements are higher when birds are infected with coccidiosis (Lai et al., 2018; Castro et al., 2020). Harms et al. (1967) showed that coccidiosis infection reduced egg production. And that, but increasing dietary levels of sulfur amino acid could mitigate the coccidia-induced decline in egg production. Murillo et al. (1976) showed similar effects in broilers, where increasing dietary levels of methionine overcame the growth suppression due to coccidiosis infection, but increasing dietary levels of methionine did not reduce coccidiosis-induced intestinal lesions or mitigate the decrease in plasma carotenoids associated with infection. In a study on methionine and cobalt, Southern and Baker (1982) showed that the reduction in feed efficiency in chicks infected with coccidiosis could be prevented with increased dietary methionine levels. Izquierdo et al. (1987) compared the ability of methionine and the anticoccidial, Narasin, for their potential to alleviate the growth depression due to coccidiosis infection. In their study, Narasin was more effective than dietary methionine in alleviating signs of infection, however, they may have failed to include a methionine level high enough to improve coccidiosis-induced growth suppression.

The need for sulfur amino acids for methyl transfer is believed to be critical for immune responses (Grimble and Grimble, 1998) and may result in the mobilization of amino acids from recruited tissues during the immune response. Indeed, dietary methionine requirements for humoral immunity to sheep red blood cells (**SRBC**) is greater than the need for growth (Tsiagbe et al., 1987b), and increased dietary methionine resulted in an earlier increase in IgY in response to an SRBC injection (Tsiagbe et al., 1987a). Lai et al. (2018) found that increasing dietary sulfur amino acid levels could stimulate the mRNA expressions of tumor necrosis factor alpha (**TNFα**), interleukin-2 (**IL-2**), and gamma interferon (**IFN-γ**) in cecum tonsil of coccidiosis-infected broilers. Hence, it is hypothesized that sulfur amino acid requirements are increased for birds infected with *Eimeria* spp.

In addition to nutritional management, strategies like anticoccidial antibiotics, chemicals, and coccidial vaccines have been widely used in the field. Anticoccidial antibiotics and chemicals delay the development of host immunity to coccidia (Chapman, 1999). The consumer push to eliminate antibiotics and chemicals, including anticoccidials from poultry feeds, creates a need for developing earlier immunity to coccidiosis. Coccidial vaccine (Witcombe and Smith, 2014) and adjunct immunotherapy (Arendt et al., 2016; Sand et al., 2016) have the capability of generating early immunity to coccidia and have been accepted as a common practice in controlling coccidiosis. Lacking in the existing literature is a recommendation for the optimal nutrition (e.g., dietary sulfur amino acid levels) to support broilers previously vaccinated with coccidiosis in preventing growth suppression and mounting an immune response to a coccidial infection. In our previous study, in broilers without coccidiosis vaccination, the growth suppression caused by coccidiosis infection could not be mitigated by further increasing dietary sulfur amino acid levels of ≥0.8% (Ren et al., 2020). In the current study, growth performance and intestinal immunity were evaluated to determine the optimal dietary sulfur amino acid levels for broilers that were vaccinated (1-day-old) and subsequently infected (14-day-old) with coccidiosis.

## MATERIALS AND METHODS

All animal protocols conducted were approved by the University of Wisconsin-Madison Institutional Animal Care and Use Committee (**IACUC** protocol No. A005392). The IACUC approvals were secured before the studies begun.

### Broilers and Diets

**Figure 1:**
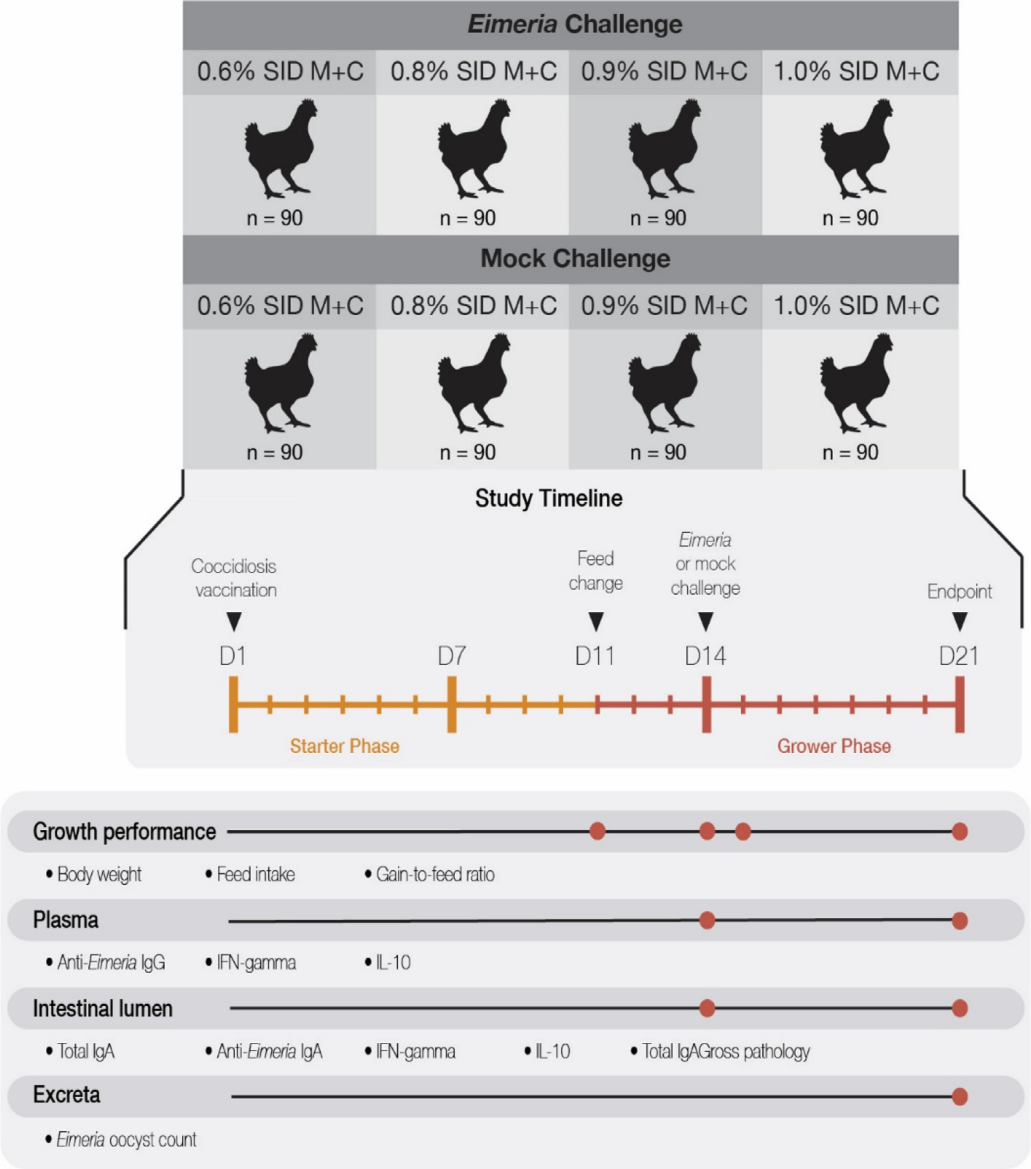
Experimental Design involved a 4 × 2 factorial arrangement of treatments. Treatments included 4 dietary standard ileal digestible methionine plus cysteine (SID M+C) levels fed to 90 broilers that all were administered *Eimeria* (coccidiosis) vaccine at 1 × on day 1 (D1) or either the *Eimeria* Challenge (vaccine at 100 ×) or a phosphate buffered saline injection (Mock challenge) on day 14 (D14). Responses were recorded at the time points indicated.

One-day-old male broilers (Ross × Ross 308; n = 720; 80 battery cages with 9 broilers in each cage; cage dimensions: length × width × height = 128 × 90 × 38 cm) were all vaccinated with an Advent (Huvepharma, Sofia, Bulgaria) coccidiosis vaccine (mixed oocysts of low virulent *Eimeria acervulina, Eimeria maxima*, and *Eimeria tenella*) at 1 × vaccine dose. From 1 to 10 d, the broilers were fed with a common starter diet formulated to meet all nutritional requirements, as shown in Table 1. The broilers had an average body weight of 327 ± 19 g (mean ± standard deviations) on d 10. On d 11, the 80 cages of broilers were assigned to 1 of 4 dietary levels of standard ileal digestible methionine plus cysteine (**SID M+C**) and 1 of 2 challenge conditions (mock vs. coccidiosis challenge) in a factorial arrangement (Figure 1). Briefly, from 11 to 21 d, the broilers were fed experimental diets containing either 0.6, 0.8, 0.9, or 1.0% of SID M+C, accomplished by adjusting the additions of subliminal DL-methionine (MetAMINO 99%, Evonik Nutrition and Care GmbH, Hanau-Wolfgang, Germany) at levels of 0, 0.2, 0.3, and 0.4%, respectively, in place of sand (River Run Products Corp., Custer, WI) as an inert ingredient (Table 1). On d 14, broilers within each of the assigned treatments were orally gavaged with either phosphate-buffered saline (Mock challenge, **PBS**; n = 10 cages from each SID M+C level) or a second injection of the Advent (Huvepharma, Sofia, Bulgaria) coccidiosis vaccine administrated at 100 × the vaccine dose as a coccidiosis challenge (*Eimeria* challenge, n = 10 cages from each SID M+C level). Compositions of the corn-soybean meal-based diets are identical to an earlier study (Ren et al., 2020) and can be found in Table 1. The formulated and analyzed amino acid levels of the 4 experimental diets were listed in Table 2.

**Table 1:** Ingredient and nutrient composition of basal diets for starter and grower phases.

Item (%, unless noted)	Starter (1–10 d)	Grower (11–21 d)			
		0.6% SID M+C	0.8% SID M+C	0.9% SID M+C	1.0% SID M+C
Corn	57.26	60.06	60.06	60.06	60.06
Soybean meal, 48% CP	35.00	31.36	31.36	31.36	31.36
Soybean oil	3.14	4.09	4.09	4.09	4.09
Dicalcium phosphate, 22%	1.76	1.63	1.63	1.63	1.63
Calcium carbonate	0.74	0.71	0.71	0.71	0.71
Sodium chloride	0.37	0.37	0.37	0.37	0.37
Choline chloride, 60%	0.10	0.10	0.10	0.10	0.10
DL-Methionine, 99%	0.31	0.06	0.26	0.36	0.46
L-Lysine sulfate, 54.6%	0.24	0.17	0.17	0.17	0.17
L-Threonine, 98.5%	0.08	0.05	0.05	0.05	0.05
Premix 1	1.00	1.00	1.00	1.00	1.00
Sand	-	0.40	0.20	0.10	-
In Total	100.00	100.00	100.00	100.00	100.00
					
Nutritional composition (calculated) 2					
AMEn (kcal/kg)	3,008	3,086	3,086	3,086	3,086
Crude protein, %	21.74	20.00	20.12	20.17	20.23
SID Methionine, %	0.59	0.33	0.33	0.33	0.33
SID Cysteine, %	0.29	0.27	0.27	0.27	0.27
SID Methionine + Cysteine, %	0.88	0.60	0.60	0.60	0.60
SID Lysine, %	1.18	1.05	1.05	1.05	1.05
SID Threonine, %	0.77	0.69	0.69	0.69	0.69

1 Supplied per kilogram of diet: copper, 15 mg; iron, 40 mg; zinc, 100 mg; manganese, 100 mg; selenium, 0.35 mg; iodine, 1 mg; vitamin A, 10,000 IU; vitamin D_3_, 5,000 IU; vitamin E, 80 IU; vitamin K, 3 mg; vitamin B_1_, 3 mg; vitamin B_2_, 9 mg; vitamin B_6_, 4 mg; vitamin B_12_, 0.02 mg; nicotinic acid, 60 mg; pantothenic acid, 15 mg; biotin, 0.15 mg; folic acid, 2 mg.

2 AMEn, nitrogen-corrected apparent metabolizable energy; SID, standardized ileal digestible. SID levels were calculated based on poultry specific digestibility coefficients provided by Evonik Nutrition and Care GmbH (Hanau, Germany).

**Table 2:** Analyzed crude protein and amino acid levels of the treatment diets^1^.

Item (%, as-is basis)	Formulated	Analyzed			
		0.6% SID M+C	0.8% SID M+C	0.9% SID M+C	1.0% SID M+C
CP	20.00	18.71	19.17	19.92	18.58
Met	0.35/0.55/0.65/0.75	0.33	0.51	0.64	0.75
Cys	0.33	0.31	0.31	0.31	0.30
Met+Cys	0.68/0.88/0.98/1.08	0.64	0.82	0.95	1.05
Lys	1.15	1.14	1.18	1.19	1.09
Thr	0.80	0.76	0.78	0.78	0.74
Arg	1.32	1.27	1.29	1.31	1.21
Ile	0.84	0.81	0.82	0.83	0.78
Leu	1.72	1.62	1.64	1.65	1.55
Val	0.93	0.89	0.90	0.92	0.86
His	0.53	0.50	0.50	0.51	0.48
Phe	0.99	0.94	0.96	0.97	0.87
Gly	0.82	0.77	0.78	0.80	0.74
Ser	0.98	0.94	0.96	0.96	0.91
Pro	1.17	1.20	1.18	1.16	1.08
Ala	1.00	0.94	0.95	0.97	0.91
Asp	2.04	1.94	1.98	2.00	1.88
Glu	3.58	3.37	3.42	3.45	3.24

1 Dietary amino acid levels were analyzed using an ion-exchange chromatography (AA analyser LC 3000, Biotronic, Maintal, Germany). CP, crude protein. SID M+C, standardized ileal digestible methionine plus cysteine.

Body weight gain (**BWG**), gain-to-feed ratio (**G:F**), and feed intake (**FI**) of the broilers were recorded and calculated for the periods from 11 to 14 d, 15 to 21 d, and 11 to 21 d. On d 14 and 21, one broiler from each cage was randomly selected and euthanized by CO_2_ inhalation followed by cervical dislocation. Blood samples were collected by cardiac puncture and plasma was separated by centrifugation at 1,000 × *g* for 20 min at 4°C (plasma was stored in a −80°C freezer until analyses). Intestinal lumen contents were collected (sampling region: duodenum, middle portion; jejunum, middle portion; ileum, middle portion; cecum, blind end) and flash frozen in liquid nitrogen (stored in a −80°C freezer until analyses). On d 21, a well-accepted lesion scoring system (Johnson and Reid, 1970; Bafundo et al., 2021) was conducted on the duodenum, jejunum, ileum, and cecum of sampled broilers to determine intestine lesion scores from 0 to 4, representing no gross lesion to severe gross lesion, respectively. On d 21, excreta samples were collected from each cage and the McMaster technique (Arendt et al., 2016) was used to count the *Eimeria* oocysts. Briefly, the excreta samples were diluted in saturated salt solution, transferred to the chambers of the McMaster slide, and counted under microscope.

### Enzyme-Linked Immunosorbent Assays

Anti-*Eimeria* IgA titer and total IgA (**T-IgA**) concentrations in intestinal contents, anti-*Eimeria* IgY titer in plasma samples, as well as the concentrations of IL-10 and interferon gamma (**IFN-γ**) in intestinal lumen and plasma samples, were analyzed using ELISA procedures described in Ren et al. (2020). Briefly 1) For intestinal lumen anti-*Eimeria* IgA titer, the plates were coated with *Eimeria* antigen which was prepared using a coccidiosis vaccine (a mixture of live *Eimeria acervulina, Eimeria maxima*, and *Eimeria tenella* oocysts; Advent, Huvepharma, Sofia, Bulgaria), the duodenum, jejunum, ileum, and cecum lumen samples were diluted to 8, 3, 5, and 7 mg protein/mL (diluted using PBS containing 1% defatted milk powder; protein concentrations were determined using a Pierce BCA Protein Assay Kit from Thermo Scientific, Rockford, IL), respectively, prior to analysis, and the secondary goat anti-chicken IgA-HRP conjugated antibody (Bethyl Laboratories, Inc., Montgomery, TX) was diluted to 1:4,000. 2) For intestinal lumen T-IgA concentrations (determined using a commercial kit E30-103 from Bethyl Laboratories, Inc. (Montgomery, TX), the plates were coated with affinity purified goat antichicken IgA antibody, the duodenum, jejunum, ileum, and cecum lumen samples were diluted to 1:1,000, 1:1,500, 1:1,000, and 1:500 (using PBS containing 1% defatted milk powder), respectively, prior to analysis, and the secondary goat antichicken IgA-HRP conjugated antibody was diluted to 1:75,000. 3) For plasma anti-*Eimeria* IgY titer, the samples were diluted to 1:200 (using a protein-free blocking buffer from Pierce, Thermo Scientific) prior to analysis, and the secondary goat anti-chicken IgY(a counterpart of mammalian IgG)-HRP conjugated antibody (Bethyl Laboratories, Inc., Montgomery, TX) was diluted to 1:4,000. 4) For the determination of IL-10 concentrations, the plasma samples were diluted to 1:500 (using a protein-free blocking buffer from Pierce, Thermo Scientific), and the duodenum, jejunum, ileum, and cecum lumen samples were diluted to 1:5, 1:4, 1:4, and 1:5 (using PBS containing 1% defatted milk powder), respectively, prior to analysis. 5) For the determination of IFN-γ concentrations, the plasma samples were diluted to 1:200 (using a protein-free blocking buffer from Pierce, Thermo Scientific), and the intestinal luminal samples were not diluted, prior to analysis.

### Statistics

All the data (except for intestine lesion scores) were subjected to a two-way (4 × 2 factorial) ANOVA analysis (SPSS 23, IBM Corp., Chicago, IL). The 2 main factors were 1) dietary SID M+C levels (0.6, 0.8, 0.9, and 1.0%); and 2) coccidiosis challenge (Mock vs. *Eimeria* challenge). Duncan's test was applied as a post hoc procedure to demonstrate the differences among treatments. Linear and quadratic analyses were conducted following the two-way ANOVA to indicate trends of the measured parameters in response to the increase of dietary SID M + C levels. Intestinal lesions were analyzed using Kruskal-Wallis test, with Dunn's test as a post hoc test for treatment comparison. The results were considered as statistically significant with a *P*-value of < 0.05.

## RESULTS

### Growth Performance

As shown in Table 3, no interactions (*P* > 0.05) between the 2 experimental factors (dietary SID levels × coccidiosis challenge) were recorded for growth performance variables. During the interval of 11 to 14 d, prior to the coccidiosis challenge, broilers fed 0.6% SID M+C had decreased (*P* < 0.05) G:F ratio when compared to broilers fed 0.8, 0.9, and 1.0% SID M+C. During the intervals of 15 to 21 d and 11 to 21 d, regardless of coccidiosis challenge, broilers fed 0.6% SID M+C had decreased (*P* < 0.05) BWG and G:F ratio when compared to broilers fed 0.8, 0.9, and 1.0% SID M+C. The G:F ratio was decreased (main effect, *P* < 0.05) by coccidiosis challenge during the intervals of 15 to 21 d and 11 to 21 d.

**Table 3:** Effects of *Eimeria* challenge and dietary sulfur amino acid levels on growth performance of broilers vaccinated with coccidiosis.

Challenge 1	SID M + C (d 11–21) 2	Body weight gain (g)			Gain to feed ratio (g:g)			Feed intake (g)		
		d 11–14	d 15–21	d 11–21	d 11–14	d 15–21	d 11–21	d 11–14	d 15–21	d 11–21
Control	0.6	134	269	403	0.65	0.52	0.56	206	521	722
	0.8	156	326	482	0.73	0.60	0.64	212	546	753
	0.9	169	332	500	0.76	0.60	0.65	223	550	769
	1.0	148	317	465	0.72	0.62	0.65	203	517	716
*Eimeria*	0.6	143	245	387	0.65	0.45	0.52	214	554	745
	0.8	161	333	494	0.74	0.57	0.62	218	581	792
	0.9	153	322	475	0.72	0.57	0.62	211	563	768
	1.0	159	304	463	0.75	0.58	0.63	212	525	732
	SEM	4	6	9	0.01	0.01	0.01	3	9	10
Main effects										
	0.6	139	257^b^	395^b^	0.65^b^	0.48^b^	0.54^b^	210	537	733
	0.8	158	330^a^	488^a^	0.73^a^	0.59^a^	0.63^a^	215	564	773
	0.9	161	327^a^	488^a^	0.74^a^	0.59^a^	0.63^a^	217	557	768
	1.0	153	311^a^	464^a^	0.74^a^	0.60^a^	0.64^a^	207	521	724
Control		152	311	463	0.71	0.58^a^	0.62^a^	211	533	740
*Eimeria*		154	301	455	0.71	0.54^b^	0.60^b^	214	556	759
		*P*-value								
SID M+C		0.123	<0.001	<0.001	<0.001	<0.001	<0.001	0.674	0.333	0.266
Challenge		0.773	0.354	0.620	0.988	0.002	0.011	0.649	0.215	0.365
Interaction		0.533	0.790	0.843	0.544	0.583	0.760	0.592	0.927	0.923

a,b Means without a common letter differ, *P* ≤ 0.05.

1 On d 14, broilers were orally gavaged with either PBS or the Advent Coccidiosis Vaccine (Huvepharma, Sofia, Bulgaria; 100 × vaccine dose; consisting of a blend of live *Eimeria acervulina, Eimeria maxima*, and *Eimeria tenella* oocysts).

2 SID M+C, standardized ileal digestible methionine plus cysteine.

### Intestinal Lesions and Fecal Oocysts

**Figure 2:**
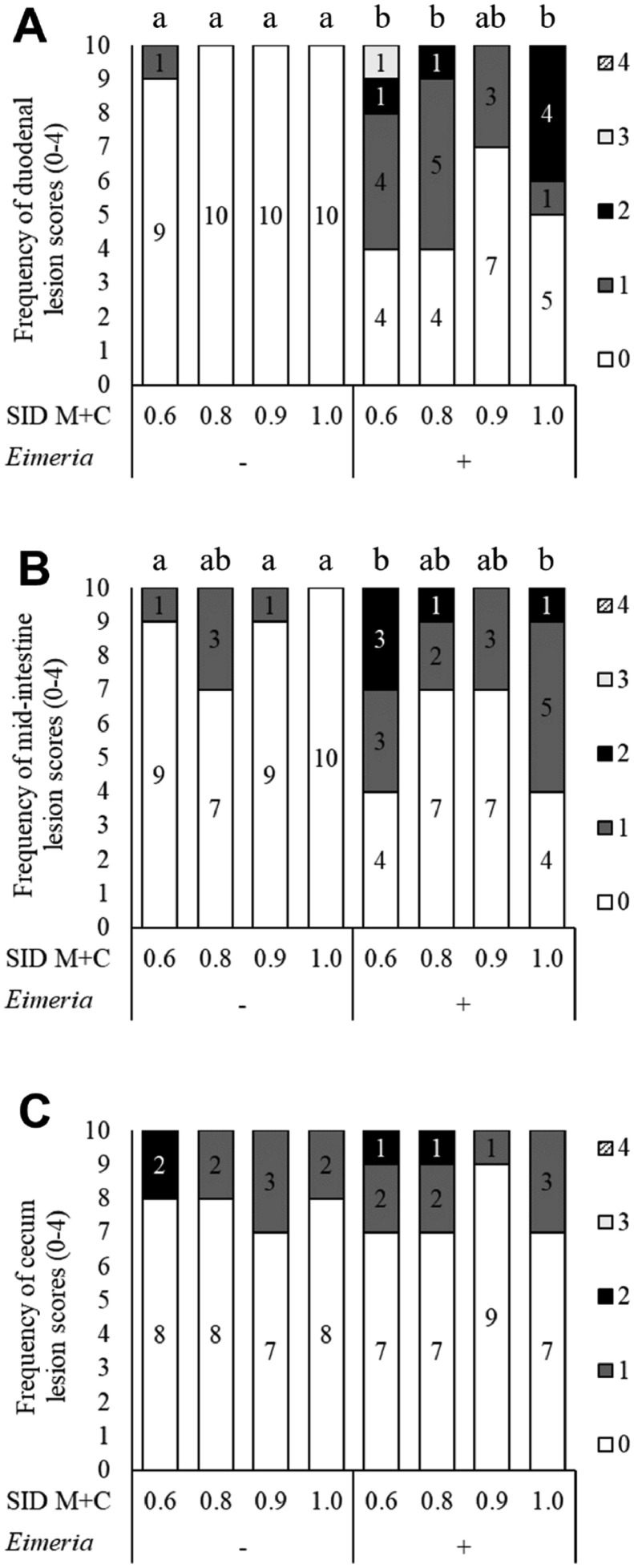
Effects of *Eimeria* challenge and dietary sulfur amino acid levels on intestinal lesion scores (A, duodenum; B, mid-intestine, i.e., jejunum and ileum; C, cecum) of broilers vaccinated with coccidiosis. On d 14, broilers were orally gavaged with either PBS or the Advent Coccidiosis Vaccine (Huvepharma, Sofia, Bulgaria; 100 × vaccine dose; consisting of a blend of live *Eimeria acervulina, Eimeria maxima*, and *Eimeria tenella* oocysts). SID M+C, standardized ileal digestible methionine + cysteine. Data are presented by the percentage of each score's category. In this study, duodenum lesion scores were ranged from 0 to 3, mid-intestine lesion scores were ranged from 0 to 2, cecum lesion scores were ranged from 0 to 2.

As shown in Figure 2, duodenum (*P* < 0.001) and mid-intestine (*P* = 0.014) lesions were significantly different among treatments. Coccidiosis challenge increased (*P* < 0.05) duodenum lesions in broilers fed 0.6, 0.8, and 1.0% SID M+C, but not in broilers fed 0.9% SID M+C (*P* > 0.05). Coccidiosis challenge increased (*P* < 0.05) mid-intestine lesions in broilers fed 0.6 and 1.0% SID M+C, but not in broilers fed 0.8 and 0.9% SID M+C (*P* > 0.05). Coccidiosis challenge increased (*P* < 0.05) fecal oocysts, regardless of dietary SID levels (Table 4).

**Table 4:** Effects of *Eimeria* challenge and dietary sulfur amino acid levels on intestinal luminal anti-*Eimeria* IgA titer and fecal oocyst counting of broilers vaccinated with coccidiosis 1.

Challenge 2	SID M + C (d 11–21) 3	d 14				d 21				
		Duodenum	Jejunum	Ileum	Cecum	Duodenum	Jejunum	Ileum	Cecum	Fecal Oocyst/g
Control	0.6	2.84	5.07	4.27	2.41	4.29	5.20	5.61	1.34	467
	0.8	3.06	5.60	4.59	2.60	4.71	5.74	6.06	2.29	1,400
	0.9	2.90	5.67	4.95	2.46	4.87	5.48	6.02	2.20	787
	1.0	3.21	5.20	4.53	2.59	4.71	5.57	5.97	1.94	860
*Eimeria*	0.6	3.03	5.33	4.40	2.69	4.47	5.62	5.61	2.09	518,847
	0.8	3.63	5.56	4.26	2.53	5.06	5.69	5.92	2.18	364,003
	0.9	2.91	5.93	4.49	2.30	4.79	5.59	6.01	2.08	374,073
	1.0	3.32	5.53	4.78	2.74	4.79	5.57	5.95	1.95	396,533
	SEM	0.13	0.10	0.10	0.06	0.07	0.07	0.09	0.13	35,493
Main effects										
	0.6	2.94	5.20	4.34	2.55	4.38	5.41	5.61	1.72	259,657
	0.8	3.34	5.58	4.42	2.57	4.89	5.72	5.99	2.23	182,702
	0.9	2.91	5.80	4.72	2.38	4.83	5.53	6.02	2.14	187,430
	1.0	3.26	5.37	4.66	2.67	4.75	5.57	5.96	1.94	198,697
Control		3.00	5.39	4.59	2.52	4.65	5.50	5.92	1.94	878^b^
*Eimeria*		3.22	5.59	4.48	2.57	4.78	5.62	5.87	2.07	413,364^a^
		*P*-value								
SID M+C		0.542	0.185	0.455	0.408	0.069	0.479	0.376	0.550	0.742
Challenge		0.400	0.324	0.602	0.673	0.362	0.397	0.810	0.629	<0.001
Interaction		0.883	0.923	0.503	0.549	0.762	0.610	0.992	0.617	0.737

1 Titer was defined as Log 2 of the highest dilution of sample with an optical density equal to the standard intestinal luminal diluted 1:128 (cutoff, two times of the background).

2 On d 14, broilers were orally gavaged with either PBS or the Advent Coccidiosis Vaccine (Huvepharma, Sofia, Bulgaria; 100 × vaccine dose; consisting of a blend of live *Eimeria acervulina, Eimeria maxima*, and *Eimeria tenella* oocysts).

3 SID M+C, standardized ileal digestible methionine + cysteine.

### Intestinal Luminal Anti-*Eimeria* IgA and T-IgA

As shown in Tables 4 and 5, on d 14 (before coccidiosis challenge), no difference (*P* > 0.05) was recorded for the intestinal luminal anti-*Eimeria* IgA and total IgA levels among treatments. On d 21 (7 d after coccidiosis challenge), no interactions (*P* > 0.05) between the 2 experimental factors (dietary SID levels × coccidiosis challenge) were recorded for intestinal luminal levels of anti-*Eimeria* IgA and T-IgA.

**Table 5:** Effects of *Eimeria* challenge and dietary sulfur amino acid levels on intestinal luminal total IgA concentrations of broilers vaccinated with coccidiosis.

Challenge 1	SID M+C (d 11–21) 2	d 14 (μg/mg protein)				d 21 (μg/mg protein)			
		Duodenum	Jejunum	Ileum	Cecum	Duodenum	Jejunum	Ileum	Cecum
Control	0.6	5.14	21.46	24.26	3.68	11.21	18.27	29.79	3.33
	0.8	5.20	18.71	29.09	3.22	10.31	17.57	34.00	4.98
	0.9	5.89	18.48	32.37	3.64	11.11	19.26	37.82	4.49
	1.0	5.43	20.07	25.63	3.31	19.11	20.09	32.58	5.40
*Eimeria*	0.6	5.17	20.99	23.80	3.61	17.73	18.61	38.55	4.63
	0.8	5.48	21.51	25.90	3.81	17.74	17.27	36.47	4.57
	0.9	5.52	17.38	29.21	3.20	18.43	18.16	39.94	3.92
	1.0	5.79	20.67	24.25	3.91	16.86	24.40	37.63	4.47
	SEM	0.23	0.92	1.24	0.32	1.32	1.03	1.44	0.23
Main effects									
	0.6	5.16	21.23	24.03	3.65	14.47	18.44	34.17	3.98
	0.8	5.34	20.11	27.49	3.52	14.03	17.42	35.24	4.77
	0.9	5.71	17.93	30.79	3.42	14.77	18.71	38.88	4.21
	1.0	5.61	20.37	24.94	3.61	17.99	22.25	35.10	4.93
Control		5.42	19.68	27.84	3.46	12.94	18.80	33.55	4.55
*Eimeria*		5.49	20.14	25.79	3.63	17.69	19.61	38.15	4.40
		*P*-value							
SID M+C		0.845	0.647	0.232	0.995	0.698	0.400	0.687	0.432
Challenge		0.882	0.812	0.419	0.802	0.077	0.700	0.124	0.747
Interaction		0.950	0.896	0.975	0.932	0.494	0.805	0.842	0.335

1 On d 14, broilers were orally gavaged with either PBS or the Advent Coccidiosis Vaccine (Huvepharma, Sofia, Bulgaria; 100 × vaccine dose; consisting of a blend of live *Eimeria acervulina, Eimeria maxima*, and *Eimeria tenella* oocysts).

2 SID M+C, standardized ileal digestible methionine plus cysteine.

### Intestinal Luminal IL-10 and IFN-γ

As shown in Table 6, no interaction between the 2 experimental factors (dietary SID M+C levels × coccidiosis challenge) was recorded for jejunal luminal IL-10 concentrations. Coccidiosis challenge increased (*P* < 0.05) luminal concentrations of IL-10 and IFN-γ in the duodenum and jejunum, regardless of dietary SID levels.

**Table 6:** Effects of *Eimeria* challenge and dietary sulfur amino acid levels on plasma and intestinal luminal IL-10 and IFN-γ concentrations of broilers vaccinated with coccidiosis (d 21)^1^.

Challenge^2^	SID M+C (d 11–21)^3^	Plasma		Duodenum		Jejunum		Ileum		Cecum	
		IL-10	IFN-γ	IL-10	IFN-γ	IL-10	IFN-γ	IL-10	IFN-γ	IL-10	IFN-γ
Control	0.6	115.21	13.89	122.70	12.16	31.84	1.91	36.03	9.11	16.81	0.75
	0.8	178.79	12.78	149.02	4.77	25.61	2.91	37.81	6.33	55.05	0.45
	0.9	127.67	12.93	112.73	11.70	31.78	2.79	41.30	7.91	35.19	0.80
	1.0	186.57	13.10	137.91	10.98	36.66	2.95	44.23	6.59	33.30	0.22
*Eimeria*	0.6	134.08	9.88	201.42	34.71	69.56	4.49	79.88	12.12	24.11	1.39
	0.8	126.86	17.05	174.68	39.45	43.25	4.27	56.99	8.29	40.40	0.64
	0.9	141.49	14.64	172.58	33.72	30.35	5.27	32.80	10.01	47.80	0.47
	1.0	117.76	13.67	195.83	31.20	37.48	4.08	38.46	6.64	92.99	1.02
	SEM	9.65	0.63	13.04	2.93	3.08	0.39	4.95	0.57	7.99	0.10
Main effects											
	0.6	124.65	11.89	162.06	22.43	50.70	3.20	57.96	10.61	20.46	1.07
	0.8	152.82	14.91	161.85	22.11	34.43	3.59	47.40	7.31	47.73	0.54
	0.9	134.58	13.79	142.66	22.71	31.07	4.03	37.05	8.96	41.49	0.64
	1.0	152.17	13.39	166.87	21.09	37.07	3.51	41.34	6.62	63.14	0.62
Control		152.06	13.17	130.59^b^	9.90^b^	31.47^b^	2.64^b^	39.84	7.49	35.09	0.55
*Eimeria*		130.05	13.81	186.13^a^	34.77^a^	45.16^a^	4.53^a^	52.03	9.26	51.33	0.88
		*P*-value									
SID M+C		0.675	0.385	0.920	0.991	0.076	0.894	0.457	0.060	0.312	0.204
Challenge		0.258	0.607	0.039	<0.001	0.018	0.017	0.217	0.117	0.315	0.096
Interaction		0.265	0.127	0.915	0.766	0.056	0.870	0.198	0.818	0.453	0.165

a,b Means without a common letter differ, *P* ≤ 0.05.

1 IL-10, interleukin-10; IFN-γ, interferon-γ. Plasma IL-10, ug/mL; plasma IFN-γ, ng/ml; intestinal IL-10, pg/mg protein; intestinal IFN-γ, ng/mg protein.

2 On d 14, broilers were orally gavaged with either PBS or the Advent ® Coccidiosis Vaccine (Huvepharma, Sofia, Bulgaria; 100 × vaccine dose; consisting of a blend of live *Eimeria acervulina, Eimeria maxima*, and *Eimeria tenella* oocysts).

3 SID M+C, standardized ileal digestible methionine plus cysteine.

### Plasma IL-10, IFN-γ, and Anti-*Eimeria* IgY

As shown in Table 6, no difference (*P* > 0.05) was recorded for plasma IL-10 and IFN-γ concentrations among treatments. However, an interaction (*P* = 0.022) between the 2 experimental factors (dietary SID M+C levels × coccidiosis challenge) was recorded on d 21 plasma anti-*Eimeria* IgY titer (Table 7). Briefly, coccidiosis challenge increased (*P* < 0.05) plasma anti-*Eimeria* IgY titer only when the broilers were fed with 0.9% SID M+C.

**Table 7:** Effects of *Eimeria* challenge and dietary sulfur amino acid levels on plasma anti-coccidia IgY titer of broilers vaccinated with coccidiosis 1.

Challenge 2	SID M+C (d 11–21) 3	d 14	d 21
Control	0.6	9.70	10.35^bc^
	0.8	9.42	10.91abc
	0.9	9.44	10.20^c^
	1.0	9.27	11.12^ab^
*Eimeria*	0.6	9.79	11.02abc
	0.8	9.67	11.02abc
	0.9	9.34	11.46^a^
	1.0	9.76	10.74abc
	SEM	0.19	0.10
Main effects			
	0.6	9.74	10.69
	0.8	9.55	10.96
	0.9	9.39	10.83
	1.0	9.51	10.93
Control		9.46	10.64^b^
*Eimeria*		9.64	11.06^a^
		*P*-value	
SID M+C		0.935	0.740
Challenge		0.647	0.033
Interaction		0.958	0.022

a,b,c Means without a common letter differ, *P* ≤ 0.05.

1 Titer was defined as Log 2 of the highest sample dilution with an optical density equal to the standard plasma diluted 1:1,600 (cutoff, two times the background).

2 On d 14, broilers were orally gavaged with either PBS or the Advent Coccidiosis Vaccine (Huvepharma, Sofia, Bulgaria; 100 × vaccine dose; consisting of a blend of live *Eimeria acervulina, Eimeria maxima*, and *Eimeria tenella* oocysts).

3 SID M+C, standardized ileal digestible methionine plus cysteine.

### Linear and Quadratic Responses of the Measured Parameters

As shown in Table 8, in mock challenge groups: the increase of dietary SID M + C levels induced linear responses of BWG (15–21 d, *P* = 0.015; 11–21 d, *P* = 0.026), G:F (11–14 d, *P* = 0.007; 15–21 d, *P* < 0.001; 11 to 21 d, *P* < 0.001), and cecum luminal total IgA (*P* = 0.045), and induced quadratic responses of BWG (15–21 d, *P* = 0.008; 11–21 d, *P* = 0.011) and G:F (11–14 d, *P* = 0.003; 15–21 d, *P* < 0.001; 11–21 d, *P* < 0.001). In *Eimeria* challenge groups: the increase of dietary SID M + C levels induced linear responses of BWG (15–21 d, *P* = 0.007; 11–21 d, *P* = 0.013), G:F (11–14 d, *P* = 0.006; 15–21 d, *P* < 0.001; 11–21 d, *P* < 0.001), jejunum luminal IL-10 (*P* = 0.014), and ileum luminal IL-10 (*P* = 0.049), and induced quadratic responses of BWG (15–21 d, *P* < 0.001; 11–21 d, *P* = 0.02), G:F (11–14 d, *P* = 0.017; 15 to 21 d, *P* < 0.001; 11–21 d, *P* < 0.001), jejunum luminal IL-10 (*P* = 0.029), and plasma IFN-γ (*P* = 0.022). Across all the experimental treatment, the increase of dietary SID M + C levels induced linear responses of BWG (15–21 d, *P* < 0.001; 11–21 d, *P* = 0.001), G:F (11–14 d, *P* < 0.001; 15–21 d, *P* < 0.001; 11–21 d, *P* < 0.001), and ileum luminal IFN-γ (*P* = 0.020), and induced quadratic responses of BWG (15–21 d, *P* < 0.001; 11 to 21 d, *P* < 0.001), G:F (11–14 d, *P* < 0.001; 15–21 d, *P* < 0.001; 11–21 d, *P* < 0.001), duodenum luminal anti-*Eimeria* IgA (*P* = 0.027), and jejunum luminal IL-10 (*P* = 0.049).

**Table 8:** Linear and quadratic analyses of the measured parameters in response to the increase of dietary SID M + C levels.^1^^,^^2^

Parameters	Linear			Quadratic		
	Mock challenge	*Eimeria* challenge	Combined	Mock challenge	*Eimeria* challenge	Combined
Body weight gain						
d 11–14	0.127	0.322	0.073	0.066	0.524	0.052
d 15–21	0.015	0.007	<0.001	0.008	<0.001	<0.001
d 11–21	0.026	0.013	0.001	0.011	0.002	<0.001
Gain to feed ratio						
d 11–14	0.007	0.006	<0.001	0.003	0.017	<0.001
d 15–21	<0.001	<0.001	<0.001	<0.001	<0.001	<0.001
d 11–21	<0.001	<0.001	<0.001	<0.001	<0.001	<0.001
Feed intake						
d 11–14	0.804	0.770	0.966	0.427	0.908	0.525
d 15–21	0.877	0.554	0.705	0.429	0.366	0.177
d 11–21	0.830	0.858	0.996	0.377	0.359	0.134
Intestinal luminal anti-*Eimeria* IgA (d 21)						
Duodenum	0.109	0.255	0.052	0.165	0.145	0.027
Jejunum	0.214	0.818	0.450	0.248	0.920	0.432
Ileum	0.364	0.214	0.124	0.504	0.387	0.191
Cecum	0.214	0.786	0.427	0.210	0.893	0.339
Intestinal luminal total IgA (d 21)						
Duodenum	0.194	0.923	0.442	0.162	0.978	0.531
Jejunum	0.611	0.293	0.258	0.810	0.232	0.232
Ileum	0.466	0.985	0.655	0.523	0.987	0.751
Cecum	0.045	0.668	0.231	0.128	0.881	0.489
Intestinal luminal IL-10						
Duodenum	0.897	0.843	0.886	0.984	0.835	0.919
Jejunum	0.440	0.014	0.051	0.228	0.029	0.049
Ileum	0.486	0.049	0.128	0.773	0.140	0.293
Cecum	0.487	0.115	0.089	0.377	0.222	0.238
Intestinal luminal IFN-γ						
Duodenum	0.985	0.779	0.717	0.544	0.853	0.935
Jejunum	0.504	0.984	0.664	0.770	0.944	0.828
Ileum	0.166	0.079	0.020	0.332	0.216	0.064
Cecum	0.304	0.179	0.089	0.519	0.066	0.131
Plasma parameters						
IL-10	0.226	0.729	0.398	0.481	0.891	0.671
IFN-γ	0.728	0.121	0.377	0.893	0.022	0.265
Anti-*Eimeria* IgY	0.244	0.821	0.443	0.476	0.461	0.676

1 Linear and quadratic analyses were conducted among mock challenge groups, among *Eimeria* challenged group, and among all the groups, respectively.

2 IFN-γ, interferon-γ; IL-10, interleukin-10; SID M+C, standardized ileal digestible methionine plus cysteine.

## DISCUSSION

In this study, the broilers were vaccinated (at 1 × vaccine dose) on d 1 to stimulate host immunity to coccidiosis and were subsequently challenged (at 100 × vaccine dose) with *Eimeria* oocysts on d 14 to induce a subclinical model of coccidiosis infection. Results of intestinal lesions and fecal oocyst shedding clearly suggest that the coccidiosis infection model had been successfully conducted. Interestingly, while coccidiosis challenge decreased the G:F ratio, no effect was observed on BW and BWG. According to our previous study (Sand et al., 2016), a 6% decrease could be expected on d 21 BW when broilers were vaccinated on d 3 (at 1 × vaccine dose) and challenged with coccidiosis (at 100 × vaccine dose) on d 17. In the current study, even though the vaccination and coccidiosis challenge were conducted in a similar manner, the coccidiosis challenge overall decreased d 21 BW of the broilers by only 0.5%, which was not a statistically significant reduction in BW. One reason that the coccidiosis challenge had less impact on growth performance than expected might be due to the dietary nutrient levels (including but not limit to amino acids, vitamins, and minerals). For example, the current experimental diets were formulated based on AMINOChick 2.0 (recommends SID M+C of 0.83% for 11- to 21-D male broilers; Evonik Operations GmbH, Hanau, Germany) amino acid recommendations which were overall higher than those used in the Sand's study (Sand et al., 2016). Indeed, the broilers in this study overall had a 23% increase in d 21 BW (800 vs. 650 g) when compared to those in Sand's study (Sand et al., 2016). These results indicate the importance of nutritional supplements during coccidiosis infection.

A parallel study was conducted in our lab (Ren et al., 2020), in which broilers were fed the same diets and reared under the same management conditions but were not vaccinated with *Eimeria* oocyst on d 1. In this former study (Ren et al., 2020), the d 15 to 21 BWG was decreased by 15, 16, 19, and 21% in broilers fed 0.6, 0.8, 0.9, and 1.0% SID M+C, respectively, after coccidiosis challenge. However, in the current study, the coccidiosis challenge decreased d 15 to 21 BWG by 9, (-)2, 3, and 7% in broilers fed 0.6, 0.8, 0.9, and 1.0% SID M+C, respectively. In the current study, we used 100 × vaccine dose for the coccidia infection. The freshness of oocysts from different batches could possibly lead to a wild range of infection severity, which might cause inconsistent infections between two studies. Otherwise, we could conclude from this comparison that d 1 vaccination effectively mitigated the growth suppression caused by coccidiosis infection. While no interaction was observed between dietary SID M+C and coccidiosis, the current results demonstrated that 0.6% SID M+C is deficient for coccidiosis-vaccinated broilers, regardless of subsequently coccidiosis challenges. This inference is consistent with conclusions in a previous study that 0.6% SID M+C was deficient for unvaccinated broilers regardless of subsequent coccidiosis challenge (Ren et al., 2020). In the current study, since the broilers were raised in battery cages (insufficient fecal-oral recycling of vaccine parasites), the immunity development after vaccination might be different from the field practice (floor pens, sufficient fecal-oral recycling of vaccine parasites). So, floor pen studies are still needed to confirm the current observations.

The intestinal barrier protects health and immune homeostasis due to a variety of immune cell types from innate and acquired immune systems (Ruth and Field, 2013). Nutrition has been linked to gut barrier function and immune status. Sulfur amino acids are essentially associated with protein synthesis for body growth (Bauchart-Thevret et al., 2009). When sulfur amino acids were supplemented in chickens at levels above those needed for growth, plasma IgY responses to phytohemagglutinin-P increased in comparison to responses if sulfur amino acids were provided at levels need to maximize growth (Tsiagbe et al., 1987b). However, the effect of sulfur amino acid on intestinal luminal antigen-specific secretory IgA (**sIgA**) production is not clear. Secretory IgA responses are seldom evaluated in studies on *Eimeria* infection as antibody responses are commonly reported as serum antibody levels. In the current study, broilers fed 0.8 and 0.9% SID M+C had increased duodenum luminal anti-*Eimeria* IgA titer on d 21 when compared to those fed 0.6% SID M+C. Similarly, in the parallel study (Ren et al., 2020), in which broilers were not vaccinated with coccidiosis on d 1, 0.8% SID M+C increased jejunum luminal anti-*Eimeria* IgA titer on d 21 when compared to 0.6% SID M+C. Lamina propria B cells in the intestines produce sIgA, the main class of secreted antibody. sIgA prevents the colonization and invasion of microbial pathogens and toxins in intestinal epithelium (Mantis and Forbes, 2010). We previously conducted an experiment to investigate how effective antibodies were for protecting against coccidiosis. *Eimeria*-challenged chicks lost 8 and 5% more BWG when compared to those challenged and orally fed an *Eimeria*-specific antibody. The chicks fed the anti-*Eimeria* antibody and then challenged, had growth equal to the unchallenged birds fed either control or anti-*Eimeria* antibodies (M. E. Cook, unpublished data). These results show that antibody at the level of the mucosa is very important immune defense against coccidiosis infection. Therefore, the increase in intestinal luminal anti-*Eimeria* sIgA titers observed in this study in relation to SID M+C levels above 0.8% may indicate protective effects against coccidiosis challenge.

Specific cytokine proteins present in the intestinal lumen may provide insights into immune mechanisms against coccidiosis infection. IFN-γ has been found to inhibit intestinal parasites (Fayer, 1971), and its increase in intestine lumen has been recognized as a host immunological strategy in response to coccidiosis challenge (Yun et al., 2000). As expected, in this study, coccidiosis challenge stimulated the production of intestinal luminal IFN-γ. The increase of intestinal luminal IL-10 in response to coccidiosis infection has been extensively repeated (Arendt et al., 2016; Sand et al., 2016). In the parallel study (Ren et al., 2020), in which broilers were not vaccinated with coccidiosis on d 1, coccidiosis challenge on d 14 stimulated intestinal luminal IL-10 production of d 21 broilers regardless of dietary SID M+C levels. Interestingly, in this study, when broilers were vaccinated (d 1) and subsequently infected (d 14) with coccidiosis, the d 21 intestinal luminal IL-10 production was increased only in those fed 0.6% SID M+C, a level that is obviously deficient for growth performance. This implies that broilers had a better intestinal immune response (lower possibility of parasite-induced IL-10 immune escape) to coccidiosis challenge with the support of both d 1 vaccination and optimal sulfur amino acid supplementation at ≥0.8% SID M+C. These results also agree with previous findings that the immunological mode of action in response to coccidiosis challenge was different between d 1 vaccinated and unvaccinated broilers (Hong et al., 2006).

While we had originally hypothesized that the requirement for sulfur amino acids to support a postvaccination coccidiosis challenge would be higher than those required for healthy unchallenged animals, the current results did not support this hypothesis, at least for growth performance. For growth variables the SID M+C level was optimal at or above 0.8% for both Mock and coccidiosis challenged groups, which aligns with current recommendations. For example, Aviagen recommends 0.87% SID M+C, while amino acid provider Evonik Operations GmbH, recommends 0.83% SID M+C. However, on d 21 (7 d after coccidiosis challenge), the highest amount of plasma anti-*Eimeria* IgY was detected in broilers vaccinated and fed 0.9% SID M+C. These immune findings do infer some improved immune protection to coccidiosis with higher dietary sulfur amino acid supplementation; however, this was not sufficient to improve growth in the challenged birds. As previously discussed in Ren et al. (2020), we hypothesize that this may be due to increased requirements for other nutrients beyond the sulfur amino acids that limited our ability to detect a response to the increased SID M+C levels. Future studies should be conducted to investigate whether an increase in other dietary factors, such as energy and other functional essential amino acids (Rochell et al., 2016, 2017; Teng et al., 2021), could synergistically improve the protective response observed to the higher levels of SID M+C in this study.

In conclusion, for broilers originally vaccinated with coccidiosis at hatch, the grower (11–21 d) dietary SID M+C requirement was ranged from 0.8 to 1.0% for optimal growth performance and intestinal immunity, regardless of coccidiosis challenge. Further studies will investigate the mechanisms of interaction between SID M+C and coccidiosis at the levels of intestinal cytokine responses and with other dietary nutrient uplifts.
